# Assessment and prevention of behavioural and social risk factors associated with oral cancer: protocol for a systematic review of clinical guidelines and systematic reviews to inform Primary Care dental professionals

**DOI:** 10.1186/s13643-015-0169-1

**Published:** 2015-12-22

**Authors:** Sweta Mathur, David I. Conway, Heather Worlledge-Andrew, Lorna M.D. Macpherson, Alastair J. Ross

**Affiliations:** Institution: University of Glasgow Dental Hospital & School, 378 Sauchiehall Street, Glasgow, G2 3JZ UK; Institution: University of Glasgow Library, Glasgow, UK

**Keywords:** Oral cavity cancer, Oropharyngeal cancer, Risk factors, Prevention, Primary care, Dental professionals, Clinical guidelines, Systematic reviews

## Abstract

**Background:**

Tobacco and alcohol are recognised as the major risk factors for both oral cavity (mouth) and oropharyngeal (throat) cancers, with increasing acceptance of the role of human papillomavirus (HPV) in the aetiology of oropharyngeal cancers. In addition, there is a significant increased risk for oral cancer among lower socioeconomic groups, males and older age groups. There is a growing evidence for the potential role of *primary care* professionals in smoking cessation and reducing alcohol-related harm. However, there are uncertainties about the best approaches/strategies to assess risk factors associated with oral cancer, effective components of preventive interventions for behaviour change and implementation strategies in primary care dental settings. Thus, in order to contribute to the prevention of oral cancer effectively, dental professionals need to assess patients on the major risk factors (tobacco, alcohol and HPV/sexual behaviours) and deliver appropriate prevention, taking into account the patient’s sociodemographic context.

**Aim:**

The study aims to synthesise evidence on the best practice for undertaking an assessment of major behavioural risk factors associated with oral cancer and delivering effective behaviour change preventive interventions (e.g. advice, counselling, patient recall, signposting/referral to preventive services) by dental professionals in primary care dental settings.

**Method:**

The study involves a systematic review and evidence appraisal. We will search for clinical guidelines and systematic reviews from the following databases: *Cochrane Library*, *Ovid MEDLINE*, *EMBASE*, *Web of Science*, *PsychINFO*, *PubMed*, *TRIP* and *Google Scholar*. We will also search websites of professional organisations/agencies and bibliographies/reference lists of selected papers. Quality will be assessed with the AGREE II (Appraisal of Guidelines for Research & Evaluation II) instrument for included clinical guidelines and the AMSTAR (A Measurement Tool to Assess Systematic Reviews) and ROBIS instruments for included systematic reviews. The best practice evidence will be assessed via a narrative synthesis of extracted data, considering publication quality.

**Discussion:**

This systematic review will synthesise evidence on the best practice for oral cancer risk factor assessment and prevention and evaluate the relationship between available clinical guidelines and the review evidence base. This collation of evidence will be useful for making recommendations for future intervention, research and guideline development.

**Systematic review registration:**

PROSPERO CRD42015025289

**Electronic supplementary material:**

The online version of this article (doi:10.1186/s13643-015-0169-1) contains supplementary material, which is available to authorized users.

## Background

### Oral cancer

The term oral cancer broadly includes oral cavity (mouth) and oropharyngeal (throat) cancers, and consensus on the definition is emerging [1]. Based on the World Health Organisation (WHO) International Statistical Classification of Diseases and Related Health Problems 10th revision (ICD-10) coding, oral cavity cancer include cancers of the lip (excluding external surface) (C00.3-C00.9), other and unspecified parts of tongue (C02, excluding C02.4), the gum (C03), floor of mouth (C04), palate (C05), other and unspecified parts of mouth (C06), but not cancers of the salivary glands. Oropharyngeal cancer include cancers of the base of tongue (C01), lingual tonsil (C2.4), tonsil (C9.0-9.9), oropharynx (C10.0-10.9), pharynx not otherwise specified (C14.0) and waldeyer ring (C14.2) [2].

The World Cancer Report 2014 has oral cavity and oropharyngeal cancers combined as the seventh most commonly occurring cancer, and in terms of mortality, they rank 9th in the world [3]. In 2012, approximately 442,760 new oral cancer cases were diagnosed, with 241,418 deaths reported worldwide [4]. The incidence of oral cavity cancer has marginally increased or stayed stable across the world, whilst oropharyngeal cancer incidence is among the most rapidly increasing in both men and women, and in younger age groups (age ˂60 years) [1]. In Scotland, oropharyngeal cancer rates are now higher than cervical cancer, melanoma of the skin and adenocarcinoma of the oesophagus [5].

Oral cancer survival is linked to stage at diagnosis [6], and there are persistent concerns about missed opportunities for early referral [7, 8]. The General Dental Council (GDC) has recently identified improved oral cancer detection as a recommended area for the Continuing Professional Development (CPD) of dental professionals [9]. Moreover, the GDC has an expectation that practitioners deliver oral cancer prevention. In a recent hearing, a dentist was placed under supervision for failing to ensure that a patient with ulceration was urgently referred to a specialist, but also for a cited charge that the dentist ‘failed to ensure that Patient A [who smoked 10-15 cigarettes a day] was provided with smoking cessation advice’ [9].

### Risk factors

Oral cancer aetiology is multifactorial and the major risk factors can be categorised as modifiable (behavioural) and non-modifiable (sociodemographic) (see Table 1) [10, 11].

**Table 1:** Major recognised oral cancer risk factors

Modifiable (behavioural)	Non-modifiable (sociodemographic)
Tobacco smoking	Socioeconomic status (lower)
Alcohol	Age (older)
Smokeless tobacco (areca/betel nut)	Gender (males)
HPV/sexual behaviours	

There is an explicit evidence establishing tobacco smoking [12] and alcohol drinking [13] as the major risk factors in oral cancer development. The risk of developing oral cancer increases with frequency (i.e. numbers of cigarettes or drinks per day or week) and duration (i.e. years of smoking or drinking) of tobacco and alcohol use [10]. In addition, oral cavity cancer risk is increased by using smokeless tobacco (snuff; chewing betel quid with or without tobacco) [10, 14], and again the risk increases with the quantity and duration of consumption [14]. Pooled data analysis from the International Head and Neck Cancer Epidemiology Consortium (INHANCE) found that the combined effects of tobacco and alcohol use are greater than the multiple of their individual effects on the risk of developing oral cancer [15]. Thus, the majority of oral cavity (64 %), pharyngeal (72 %), and laryngeal cancers (89 %) are associated with these behaviours combined [15]. Another INHANCE study concluded that a beneficial effect on reducing oral cancer risk was observed following smoking cessation and quitting alcohol drinking [16].

Recent evidence from studies conducted in the USA shows increased oral cancer rates, particularly oropharyngeal cancer, which are thought to be partly attributable to the human papillomavirus (HPV) and especially HPV16 which is sexually transmitted [17, 18]. An INHANCE analysis found an increased oropharyngeal cancer risk associated with certain sexual behaviours, i.e. history of having six or more sexual partners, four or more oral sex partners, an earlier age at sexual debut and same-sex sexual contact [17].

In addition to behavioural risk factors, the major non-modifiable risk factors include socioeconomic status, age and gender [10, 19]. According to a large INHANCE pooled data analysis, lower education status and lower income have been implicated as important oral cancer risk factors, independent of behavioural risk factors [20]. Moreover, oral cancer is more common in males than in females, with two thirds of oral cavity cancer cases occurring in men worldwide [4]. Whilst there is also an increased risk for oral cancer among older age groups, with the majority of cases occurring in people aged 50 or over [4]. A further INHANCE study compared the role of major oral cancer risk factors (tobacco smoking and alcohol drinking) in younger adults and older adults and found a positive association with oral cancer and these major risk factors, independent of age [21].

### Prevention

Following the identification of risk, preventive interventions available to dental professionals include advice, behavioural counselling, adjusted patient recall intervals and/or signposting/referral to preventive services. The main current focus of preventive strategies is the delivery of a ‘brief intervention’ , which is defined as 5-15 min of motivational and/or supportive discussion akin to basic counselling [22]. For example in Scotland, the Government’s smoking and alcohol strategy includes the delivery of brief interventions through *primary care* professionals and outlines the cost-effectiveness of these interventions, especially when combined with referral to specialist cessation services [23, 24]. However, it is often still hard to establish which components of interventions are most effective, particularly in dental practice settings [25].

Given the growing acceptance of the role of HPV in the aetiology (and HPV testing in the management) of oropharyngeal cancers, the implications for patient/social history taking in oral health assessment and for patient counselling needs to be considered [26]. For example, according to the Centers for Disease Control and Prevention HPV guidelines, behavioural counselling on safer sexual practices (i.e. lifetime mutual monogamy, condom use and fewer sexual partners) plays an important role in oropharyngeal cancer prevention [27].

### Common and multiple risk factors

The behavioural risk factors implicated in oral cancer risk are also associated with a wide range of diseases (such as heart disease, cancer, diabetes, stroke and other oral diseases); hence tobacco, alcohol and socioeconomic status in particular are known as common risk factors [28]. The associated common risk factor approach recognises that dental professionals can contribute to improve not only oral health but also general health. The WHO has supported this approach at a global level, and an effective oral cancer prevention strategy may have benefits that are not limited to this particular condition alone [28, 29].

Consideration also needs to be given to focusing on the presence of *multiple* risk factors in individuals, i.e. clustering of unhealthy behaviours and socioeconomic factors. Research has shown that risk factors (for example, smoking, alcohol, poor diet and physical inactivity) occur in combinations and show multiplicative interactions and are strongly associated with poorer socioeconomic environments [30]. This is particularly relevant to oral cancer prevention where there is a synergistic relationship between multiple risk factors, with smoking and alcohol in combination magnifying the risk for oral cancer [15].

Communicating risks associated with oral cancer is a key challenge for dental professionals and can help in changing behaviour and/or improving patient decision-making [31]. One of the strategies to promote positive health behaviour change is to identify/create a ‘teachable moment’ , which is an opportunity to implement preventive interventions [32]. There is a need to determine which teachable moments are present (or could be created) to allow dental professionals to provide effective oral cancer risk communication, and therefore should be recommended the best practice [31, 32].

There is a plethora of guidance and recommendations available for dental professionals regarding the causes of oral cancer worldwide [10, 33] and also for the early detection, effective treatment and palliative care of oral cancer cases [33]. The current evidence also shows the importance of dental professionals in providing preventive advice. However, initial literature searching, plus detailed discussions with specialists in Oral Medicine, Dental Public Health, the International Head and Neck Cancer Epidemiology Consortium (INHANCE), Postgraduate Dental Education specialists (NHS Education Scotland) and Clinical Effectiveness groups identified potential uncertainties [34] about the best approaches/strategies to assess risk factors associated with oral cancer [33], effective components of preventive interventions for behaviour change (i.e. what are the ‘Active ingredients’/mechanisms) [25, 33, 35], and implementation strategies (i.e. ‘how to do’ rather than ‘what to do’) [36] in the primary care dental settings.

This established a clear need to more clearly present details of evidence-based approaches for risk factor assessment and prevention for dental professionals for effective behaviour change to benefit those at the highest risk of oral cavity and oropharyngeal cancer (e.g. tobacco cessation, reduced alcohol consumption and safer sexual practices). Thus, this systematic review involves identifying and appraising evidence for the best practice in oral cancer risk assessment and the delivering of preventive interventions for effective behaviour change. It does not cover screening and oral examination (e.g. for early detection).

### Aims/objectives

The study aims to synthesise evidence on the best practice for undertaking an assessment of major behavioural risk factors associated with oral cancer and delivering effective behaviour change preventive interventions (e.g. advice, counselling, patient recall, signposting/referral to preventive services) by dental professionals in primary care dental settings.

Specific research questions for this systematic review in accordance with the PICOS (participants, intervention, comparator, outcomes, and setting) format are [37, 38] the following:

1. What methods for assessing major behavioural risk factors associated with oral cancer delivered by dental professionals on patients visiting primary care dental settings are considered the best practice and what are recommendations for associated preventive interventions, including advice, counselling, patient recall and signposting/referral to preventive services?
2. What methods for delivering preventive interventions for major behavioural risk factors associated with oral cancer by dental professionals on patients visiting primary care dental settings are considered the best practice for effective behaviour change?

Dental professionals here includes dentists, dental therapists, dental hygienists, dental nurses and oral health educators in primary care dental settings (i.e. the first point of contact in the health care system e.g. general dental practice and the public dental service; excluding secondary and tertiary care settings). Identifying the best practice means appraising approaches/strategies that are best supported in terms of evidence for their effectiveness and efficiency [39].

## Method

This systematic review is registered with PROSPERO (registration number CRD42015025289). The PRISMA-P 2015 statement for systematic review protocols has been consulted for writing this protocol [37, 38]. The PRISMA-P 2015 checklist is provided in Additional file 1. Other similar systematic reviews of reviews and/or guidelines have been referred to for convention, e.g. Álvarez-Bueno et al. [40], Koes et al. [41], Al-Ansary et al. [42] and Damery et al. [43].

### Eligibility criteria

#### Types of studies

Clinical guidelines (published/e-learning) and systematic reviews or meta-analyses (of randomised and non-randomised studies) available worldwide will be included in this systematic review. The included clinical guidelines, recognised by a national governmental or provider organisation, will comprise recommendations or the best practice related to risk factors associated with oral cancer, history taking, patient recall and delivery of preventive interventions in the primary care (medical and dental) setting. The included systematic reviews will contain evidence for the effectiveness of interventions for addressing risk factors associated with oral cancer and/or for preventive interventions (delivered in the primary care setting).

We will not apply any language restrictions for identifying clinical guidelines and systematic reviews. The non-English papers will be translated to English with the help of the University Translation Services department. Clinical guidelines will be limited to the last 10 years. There will be no date restrictions for systematic reviews. Narrative/literature reviews and systematic review protocols will be excluded.

#### Types of participants

Study participants/target groups include patients or subjects of any age attending primary care who are at risk of developing oral cavity or oropharyngeal cancers, i.e. tobacco users, alcohol drinkers and those at risk of developing HPV (e.g. history of having six or more sexual partners, four or more oral sex partners, early onset of sexual activity or same-sex sexual contact [17]).

#### Types of interventions

The review covers interventions by primary care professionals to assess risk and/or promote behaviour change. Interventions could include advice, brief interventions, behavioural counselling, patient recall and referral to preventive services or any combination of these. We will exclude screening interventions for the detection of oral cancer (e.g. visual screening, visual staining using toluidine blue, oral cytology using brush biopsies and fluorescence imaging and light-based techniques).

#### Types of outcome measures

The main outcomes of interest will be the following:

- The best practice in oral cancer risk factor assessment (e.g. how best to ask about behaviour/assess risk/communicate risk)
- The best practice in preventive interventions:
- Description of evidence-based preventive interventions (e.g. length and content of preventive interventions, number of follow-up sessions, referral pathways)
- Evidence for effectiveness of interventions, i.e. changes in behaviours (e.g. decrease in tobacco or alcohol consumption from baseline to follow-up)
- Role of specific aspects such as combination interventions (addressing multiple risks)

### Types of setting

The study includes primary care (medical and dental) settings; secondary or tertiary care are not included in this study.

### Information sources

The literature search for clinical guidelines and systematic reviews will be carried out in health and psychological electronic databases: *Cochrane Library*, *Ovid MEDLINE*, *EMBASE*, *Web of Science*, *PsychINFO*, *PubMed*, *TRIP* and *Google Scholar*. An internet search of the websites of health boards and relevant (professional, medical, dental, public health, scientific) organisations/agencies will also be carried out. A list of organisations/databases for searching clinical guidelines has been provided in Additional file 2. A dedicated University Librarian (HW-A) will help to develop a bespoke protocol to allow identification of clinical guidelines (published/e-learning) via websites of relevant organisations/agencies and an Internet Search Engine (Google). The bibliographies/reference lists of identified documents will also be hand-searched for additional references. Experts in the area have been contacted and will help locate any unpublished and ongoing research as the review proceeds in order to minimise publication bias.

### Search strategy

The University Librarian (HW-A) has helped develop the search strategy for identifying clinical guidelines and systematic reviews. The initial search terms were identified from a scoping of literature and from MeSH subject headings. In order to find all relevant data, the initial search terms included are broad, drawing from across primary care (both medical and dental practice setting). Evidence will only be restricted to dental practice setting if sufficient quality and quantity can be identified. Key terms are prevention (e.g. advice, cessation, harm reduction, brief intervention, counselling); primary care (e.g. General Dental Practice, General Medical Practice) and risk factors (e.g. alcohol, tobacco, HPV). Various truncation symbols (for e.g. *, ?, $) and Boolean operators (for e.g. AND, OR, proximity) will be used to refine the search. We decided not to limit the search to oral cancer, because of not wanting to rule out good guidelines and/or evidence on how to assess risk and deliver oral cancer prevention that can be extrapolated from guidelines and/or reviews aimed at another clinical condition (e.g. smoking cessation strategies targeting periodontal disease) [44].

The InterTASC Information Specialists’ Sub-Group (ISSG) search filter resource provides easy access to published and unpublished search filters designed to retrieve records by study design or focus [45]. Thus, in order to retrieve systematic reviews in *Ovid MEDLINE* and *EMBASE*, the *SIGN* search filter will be used; whereas to retrieve clinical guidelines in *Ovid MEDLINE*, the *University of Texas School of Public Health* search filter will be used [45]. The search will be limited to clinical guidelines and systematic reviews by using the filters provided in the databases *Cochrane Library*, *PsychINFO*, *PubMed and TRIP*. In the *Web of Science* database, the search will be limited to ‘Reviews’ (including literature reviews and systematic reviews), as there is no filter for systematic review only. Using search filters will help in reducing the number of articles to be screened whilst identifying higher quality evidence and maximising specificity [46].

An example of the *Ovid MEDLINE* search strategy is provided in Additional file 3. The *MEDLINE* search strategy will be finalised first and then it will be adapted for other databases. The *SIGN* search filters and the *University of Texas School of Public Health* search filters have been provided in Additional file 4.

### Data management

In accordance with the Cochrane review group guideline, all steps in data management (review of titles and abstracts, inclusion and exclusion decisions, data extraction, quality appraisal, assessing risk of bias, collating themes for final synthesis) will be carried out independently by at least two members of the multidisciplinary review team and discrepancies discussed with the wider team [47]. If, after discussions between the review team, uncertainties still persist, authors of the original studies will be contacted to resolve disagreements.

Records from all searches will be combined, imported into *EndNote* bibliographic software and duplicate records will be removed. Two investigators will independently review the title and abstract of all records against the inclusion/exclusion criteria to select for full text review. Disputed papers will be included at this stage. Full text copies of selected clinical guidelines and systematic reviews will be obtained and used to assess the eligibility of the records to be included in the final systematic review. Reasons for exclusion will be recorded and reported. Where more than one eligible clinical guideline or systematic review of the same research data exists, we will include the most recent publication.

To summarise, a PRISMA four-phase flow diagram will be completed at the end of the study selection process. The next steps will be to extract data from the clinical guideline and systematic review strands for separate synthesis and systematic quality assessment/risk of bias exercises and finally to compare and contrast to give an overview across the two streams.

### Data extraction

The data extraction template will be adapted from the *Cochrane Collaboration* [47] and the *Centre for Reviews and Dissemination* (*CRD*) *at the University of York* [48] data extraction checklist. The data extraction form will be pilot-tested on a small set of papers and refined to ensure the correct sensitivity and specificity.

Details of information to be extracted from reviews and guidelines, respectively, are provided in Additional file 5.

### Quality assessment and risk of bias

#### Clinical guidelines

Quality assessment of the included clinical guidelines will be carried out using the AGREE II (Appraisal of Guidelines for Research & Evaluation II) instrument. This consists of 23 key items in six domains and will be used to assess the methodological rigour and transparency in the development of the included clinical guidelines [49]. Each domain helps to appraise the quality of clinical guideline in a unique dimension, i.e. scope and purpose, stakeholder involvement, rigour of development, clarity of presentation, applicability and editorial independence. This instrument also assigns each clinical guideline an overall quality rating between 1 (lowest possible quality) and 7 (highest possible quality), and whether the user would recommend the guideline for use in practice or not.

#### Systematic reviews

In order to assess the methodological quality of included systematic reviews, the AMSTAR (A Measurement Tool to Assess Systematic Reviews) instrument will be used [50]. The AMSTAR checklist consists of 11 key items designed to help systematically rate the quality of various methodological aspects (e.g. an ‘a priori’ design, data extraction by at least two independent reviewers, at least two electronic sources searched, duplicate study selection and assessment of publication bias) [50]. In addition, the ROBIS tool will be used for assessing the risk of bias in included systematic reviews [51]. This is a new tool which is completed by assessing relevance, identifying concerns with the review process and finally judging risk of bias.

### Data synthesis

We will first keep the clinical guidelines and systematic reviews as separate ‘streams’, and each will be synthesised rigorously according to guidance on conducting systematic reviews [48]. Because included clinical guidelines and systematic reviews are likely to be heterogeneous in relation to included interventions, target populations, methods, etc., we will focus on a thematic description of each stream in line with our objectives.

The main steps in synthesis will be extraction and organisation of data, analysis between and within risk factors and professional groups and assessing robustness of evidence and recommendations [48, 52, 53]. As an integral component of each narrative synthesis, data will be related to the quality assessment in order to illustrate the strongest evidence among included clinical guidelines and systematic reviews [53].

Tables including a full description of included studies (e.g. study quality, risk factors assessed, included interventions, study population and outcomes) will be presented to summarise the evidence. This will be followed by a narrative description of the study characteristics presented in the tables, considering the methodological biases and other problems affecting the interpretation of study outcomes. Heterogeneity or sources of variability among study populations, settings, or outcomes will be explored as an integral part of data synthesis, but as this work is not meta-analytic, we will use the narrative synthesis to address the applicability of findings across, for example, professional groups and/or patient behaviours. A list of excluded studies will also be presented with the reasons for exclusion.

We will follow the general framework for conducting narrative synthesis developed by Popay et al. i.e. Economic and Social Research Council (ESRC) Methods Programme [52], CRD [48] and Petticrew and Roberts [53]. These frameworks will be adapted for evidence synthesis from clinical guidelines.

#### Overview of clinical guidelines and systematic reviews

After this within-stream synthesis, we then want to ask specific questions about whether evidence from reviews is reflected in current guidance or whether collated guidance shows areas where better evidence is required. Whilst our appraisal and extraction methods follow validated protocols, the ‘higher level’ synthesis of these two streams in this way is innovative, and we believe will be a good contribution to knowledge. We will establish the best evidence for risk assessment and/or preventive interventions and then examine carefully the link between this review evidence and clinical guidelines. This will allow us to make recommendations where (a) gaps in guidance are identified or (b) guidance shows areas where more evidence is required.

## Discussion

This systematic review will identify the best practice for oral cancer risk factor assessment and preventive interventions for dental professionals in primary care dental settings. This study will consider preventive interventions around common behavioural risk factors (tobacco, alcohol and HPV/sexual behaviours), recognising the role of sociodemographic factors. The collation of this evidence will help to identify gaps in the available clinical guidelines and the evidence base and gaps in evidence overall. The study will form the basis for informing the profession in this important field, by helping design future interventions, research and guidelines where necessary.

This study is novel in synthesising evidence from both clinical guidelines and systematic reviews. After conventional synthesis of each stream, we will develop methods for evidence synthesis across these information sources. Another strength of this study is that the systematic search is not limited to oral cancer, thus we do not rule out good guidelines and/or evidence on how to assess risk and deliver prevention for these risk factors that may be aimed at another clinical condition (e.g. periodontal disease) [44]. Whilst our broad coverage of both guidelines and reviews will give a good overview of this important area, heterogeneity in document type and/or evidence extracted might limit the ability to make conclusive recommendations regarding effective components that could be delivered by dental professionals in regular patient visits.

### Dissemination of findings

Findings will be reported using the PRISMA statement for reporting of systematic reviews and meta-analyses [54]. The findings will be submitted as part of a thesis for PhD degree. We will also submit the findings for publication in a relevant peer-reviewed journal and present findings in scientific meetings/conferences.

## Additional files

Additional file 1:
**The PRISMA-P 2015 checklist for systematic review protocols has been completed and uploaded.** (DOC 83 kb) Additional file 2:
**A list of organizations/databases for searching clinical guidelines has been uploaded.** (DOCX 13.2 kb) Additional file 3:
**A sample MEDLINE search strategy has been uploaded (it will be adapted for other database searches).** (DOCX 12.3 kb) Additional file 4:
**Search filters (SIGN and the University of Texas School of Public Health) to identify systematic reviews and clinical guidelines has been uploaded.** (DOCX 14.7 kb) Additional file 5:
**Data extraction form- details of information to be extracted from systematic reviews and clinical guidelines has been uploaded.** (DOCX 20.7 kb)

## Abbreviations

AGREE II: Appraisal of Guidelines for Research & Evaluation II
AMSTAR: A Measurement Tool to Assess Systematic Reviews
CPD: Continuing Professional Development
CRD: Centre for Reviews and Dissemination
EMBASE: Excerpta Medica Database
ESRC: Economic and Social Research Council
GDC: General Dental Council
HPV: Human Papillomavirus
ICD-10: International Statistical Classification of Diseases and Related Health Problems 10th revision
INHANCE: International Head and Neck Cancer Epidemiology Consortium
ISSG: InterTASC Information Specialists’ Sub-Group
MEDLINE: Medical Literature Analysis and Retrieval System Online
MeSH: Medical Subject Headings
NHS: National Health Service
NICE: National Institute for Health and Care Excellence
PICOS: Participants, Intervention, Comparator, Outcomes, and Setting
PRISMA: Preferred Reporting Items for Systematic Reviews and Meta-Analyses
PRISMA-P: Preferred Reporting Items for Systematic Review and Meta-Analysis Protocols
PROSPERO: International Prospective Register of Systematic Reviews
ROBIS: tool to assess risk of bias in systematic reviews
SDCEP: Scottish Dental Clinical Effectiveness Programme
SIGN: Scottish Intercollegiate Guidelines Network
TRIP: Turning Research Into Practice
WHO: World Health Organisation
