# Supplementary material for: Assessment and prevention of behavioural and social risk factors associated with oral cancer: protocol for a systematic review of clinical guidelines and systematic reviews to inform Primary Care dental professionals
Source: Syst Rev. 2015 Dec 22;4:184. doi: 10.1186/s13643-015-0169-1 (PMC4689050; doi:10.1186/s13643-015-0169-1)
Supplement: Additional file 2: — A list of organizations/databases for searching clinical guidelines has been uploaded. (DOCX 13.2 kb) [file 13643_2015_169_MOESM2_ESM.docx]

**Additional file 2: List of organizations/databases for searching clinical guidelines**

|  | **Organizations/Databases** | **Website** |
| --- | --- | --- |
| 1 | British Dental Association | http://www.bda.org/ |
| 2 | British Society for Oral Medicine | http://www.bsom.org.uk/ |
| 3 | British Association of Head & Neck Oncologists | http://www.bahno.org.uk/ |
| 4 | National Institute for Health and Care Excellence | http://www.nice.org.uk/ |
| 5 | General Dental Council | http://www.gdc-uk.org/ |
| 6 | Scottish Intercollegiate Guidelines Network | http://www.sign.ac.uk/ |
| 7 | Cancer Research UK | http://www.cancerresearchuk.org/ |
| 8 | Health Technology Assessment | http://www.hta.ac.uk/ |
| 9 | European Association of Oral Medicine | http://www.eaom.eu/ |
| 10 | Mouth cancer foundation | http://www.mouthcancerfoundation.org/ |
| 11 | Centers for Disease Control and Prevention | http://www.cdc.gov/ |
| 12 | College of Dietitians of British Columbia | http://www.collegeofdietitiansofbc.org/ |
| 13 | American cancer society | http://www.cancer.org/ |
| 14 | US Preventive Services Task Force | http:// www.uspreventiveservicestaskforce.org/ |
| 15 | World Health Organization | http://www.who.int/ |
| 16 | EUROPA - European Union website | http://europa.eu/ |
| 17 | New Zealand Guidelines Group | http://www.health.govt.nz/about-ministry/ministry-health-websites/new-zealand-guidelines-group |
| 18 | Agency for Healthcare Research and Quality (AHRQ) | www.ahrq.gov/professionals/clinicians-providers |
| 19 | American College of Physicians Clinical Practice Guidelines | www.acponline.org/clinical_information/guidelines/guidelines/ |
| 20 | ADA Center of Evidence-Based Dentistry | www.ebd.ada.org |
| 21 | Australian National Health and Medical Research Council | https://www.clinicalguidelines.gov.au/ |
| 22 | Institute for Clinical Systems improvement | https://www.icsi.org/guidelines__more/ |
