# Supplementary material for: Assessment and prevention of behavioural and social risk factors associated with oral cancer: protocol for a systematic review of clinical guidelines and systematic reviews to inform Primary Care dental professionals
Source: Syst Rev. 2015 Dec 22;4:184. doi: 10.1186/s13643-015-0169-1 (PMC4689050; doi:10.1186/s13643-015-0169-1)
Supplement: Additional file 3: — A sample MEDLINE search strategy has been uploaded (it will be adapted for other database searches). (DOCX 12.3 kb) [file 13643_2015_169_MOESM3_ESM.docx]

**Additional file 3: MEDLINE search strategy**

1. (primary adj3 prevention*).mp.

2. Primary Prevention/ or "primary prevention*".mp.

3. Counseling/ or Patient Education as Topic/ or advice*.mp.

4. "Tobacco Use Cessation"/ or cessation*.mp. or Smoking Cessation/

5. Harm Reduction/ or "harm reduction*".mp.

6. (harm adj3 reduction*).mp.

7. Psychotherapy, Brief/ or "brief intervention*".mp.

8. (brief adj3 intervention*).mp.

9. Early Medical Intervention/ or intervention*.mp.

10. "early intervention*".mp.

11. "minimal intervention*".mp.

12. "general pract* intervention*".mp.

13. "brief counsel?ing".mp.

14. (brief adj3 counsel?ing).mp.

15. "behavio?r* counsel?ing".mp.

16. (behavio?r* adj3 counsel?ing).mp.

17. Sex Counseling/ or "sex* counsel*".mp.

18. Communication/ or Motivational Interviewing/ or "brief communication".mp.

19. "alcohol reduction*".mp.

20. "control* drink*".mp.

21. "health promotion".mp. or Health Promotion/

22. Risk Assessment/ or "risk assess*".mp.

23. "patient recall".mp.

24. referral.mp. or "Referral and Consultation"/

25. signpost*.mp.

26. 1 or 2 or 3 or 4 or 5 or 6 or 7 or 8 or 9 or 10 or 11 or 12 or 13 or 14 or 15 or 16 or 17 or 18 or 19 or 20 or 21 or 22 or 23 or 24 or 25

27. Primary Health Care/ or "primary care*".mp.

28. (primary adj3 care*).mp.

29. General Practice/ or Family Practice/ or "general practice*".mp.

30. (general adj3 practice*).mp.

31. "medical practice*".mp.

32. General Practice, Dental/ or Dental Care/ or "dental practice*".mp.

33. Dental Clinics/ or "dental clinic*".mp.

34. "dental setting*".mp.

35. Dental Offices/ or "dental office*".mp.

36. "community care*".mp. or Community Health Services/

37. Patient Care/ or "patient care*".mp.

38. "shared care*".mp.

39. "clinical care".mp.

40. 27 or 28 or 29 or 30 or 31 or 32 or 33 or 34 or 35 or 36 or 37 or 38 or 39

41. Alcohol Drinking/ or alcohol*.mp.

42. "Tobacco Use"/ or Tobacco/ or Tobacco, Smokeless/ or tobacco*.mp. or Tobacco Products/

43. Smoking/ or smok*.mp.

44. cigar*.mp.

45. Areca/ or quid*.mp.

46. snuff*.mp.

47. HPV.mp. or Human papillomavirus 16/ or Papillomavirus Infections/

48. "wart* virus*".mp.

49. 41 or 42 or 43 or 44 or 45 or 46 or 47 or 48

50. 26 and 40 and 49
