# Supplementary material for: Assessment and prevention of behavioural and social risk factors associated with oral cancer: protocol for a systematic review of clinical guidelines and systematic reviews to inform Primary Care dental professionals
Source: Syst Rev. 2015 Dec 22;4:184. doi: 10.1186/s13643-015-0169-1 (PMC4689050; doi:10.1186/s13643-015-0169-1)
Supplement: Additional file 4: — Search filters (SIGN and the University of Texas School of Public Health) to identify systematic reviews and clinical guidelines has been uploaded. (DOCX 14.7 kb) [file 13643_2015_169_MOESM4_ESM.docx]

**Additional file 4: Search filters to identify systematic reviews and clinical guidelines**

1. **SIGN search filters to identify systematic reviews or meta-analyses**

| **MEDLINE** | **EMBASE** |
| --- | --- |
| 1. Meta-Analysis as Topic/  2. meta analy$.tw.  3. metaanaly$.tw.  4. Meta-Analysis/  5. (systematic adj (review$1 or overview$1)).tw.  6. exp Review Literature as Topic/  7. or/1-6  8. cochrane.ab.  9. embase.ab.  10. (psychlit or psyclit).ab.  11. (psychinfo or psycinfo).ab.  12. (cinahl or cinhal).ab.  13. science citation index.ab.  14. bids.ab.  15. cancerlit.ab.  16. or/8-15  17. reference list$.ab.  18. bibliograph$.ab.  19. hand-search$.ab.  20. relevant journals.ab.  21. manual search$.ab.  22. or/17-21  23. selection criteria.ab.  24. data extraction.ab.  25. 23 or 24  26. Review/  27. 25 and 26  28. Comment/  29. Letter/  30. Editorial/  31. animal/  32. human/  33. 31 not (31 and 32)  34. or/28-30,33  35. 7 or 16 or 22 or 27  36. 35 not 34 | 1. exp Meta Analysis/  2. ((meta adj analy$) or metaanalys$).tw.  3. (systematic adj (review$1 or overview$1)).tw.  4. or/1-3  5. cancerlit.ab.  6. cochrane.ab.  7. embase.ab.  8. (psychlit or psyclit).ab.  9. (psychinfo or psycinfo).ab.  10. (cinahl or cinhal).ab.  11. science citation index.ab.  12. bids.ab.  13. or/5-12  14. reference lists.ab.  15. bibliograph$.ab.  16. hand-search$.ab.  17. manual search$.ab.  18. relevant journals.ab.  19. or/14-18  20. data extraction.ab.  21. selection criteria.ab.  22. 20 or 21  23. review.pt.  24. 22 and 23  25. letter.pt.  26. editorial.pt.  27. animal/  28. human/  29. 27 not (27 and 28)  30. or/25-26,29  31. 4 or 13 or 19 or 24  32. 31 not 30 |

1. **University of Texas School of Public Health search filters to identify guidelines/recommendations**

| **MEDLINE** |
| --- |
| practice guideline/ or Health Planning Guidelines/ or guideline*.ti. or (practice adj3 parameter*).ti,ab. or clinical protocols/ or guidance.ti,ab. or care pathway*.ti,ab. or critical pathway/ or (clinical adj3 pathway*).ti,ab. or algorithms/ or consensus development conference.pt. or consensus development conference nih.pt. |
