# Supplementary material for: Assessment and prevention of behavioural and social risk factors associated with oral cancer: protocol for a systematic review of clinical guidelines and systematic reviews to inform Primary Care dental professionals
Source: Syst Rev. 2015 Dec 22;4:184. doi: 10.1186/s13643-015-0169-1 (PMC4689050; doi:10.1186/s13643-015-0169-1)
Supplement: Additional file 5: — Data extraction form- details of information to be extracted from systematic reviews and clinical guidelines has been uploaded. (DOCX 20.7 kb) [file 13643_2015_169_MOESM5_ESM.docx]

**Additional file 5: Data extraction form**

1. **Details of information to be extracted from Systematic Reviews**

| 1. **Identification features**  - Record number/code (to uniquely identify study) - Author - Organization (e.g. Journal, Cochrane, etc.) - Article title - Citation - Source of Funding - Site (e.g. country)      1. **Review characteristic**  - Aims/objectives/research questions - Number of included studies - Types of study included (e.g. systematic review, RCT, quasi-experimental, etc.) - Type of synthesis (e.g. narrative synthesis, meta-analysis) - Sources searched (e.g. databases, organizations, health boards, etc.) - Data collection period (range/years included, e.g. 1950-2000) - Inclusion and exclusion criteria (e.g. any language restrictions, etc.) - Country of origin of included studies - Appraisal instruments used and appraisal rating (e.g. AMSTAR, AGREE II, etc.)      1. **Participant characteristics**  - Age group - Gender - Socioeconomic status - Tobacco user, alcohol user, HPV / sexual behaviours, etc.  1. **Interventions**  - Description of preventive intervention (e.g. aim; behavioural/pharmacologic; length and content of intervention; delivered by whom; number of follow up sessions; referral pathways) - Risk factors assessed or focused on (e.g. tobacco, alcohol, HPV, etc.) - Method of risk factor assessment (e.g. how to ask about behaviour / assess risk) - Different options for assessment/prevention of the condition or health issue   **Comparison**   - v/s no preventive intervention and comparison in high risk group  1. **Outcome data/results**  - Pre-specified outcomes (primary and secondary) - Evidence for behaviour change/ effectiveness of intervention (e.g. decrease in tobacco or alcohol consumption from baseline to follow-up, reduce mortality, length of follow up) - Role of combined interventions (e.g. multiple risks) - Role of communicating risks (e.g. identifying teachable moment) - Adverse events - **Settings/context** - Type of practice- Primary care setting- medical or dental   **Main results and authors conclusions**  **Summary points/Notes**  Any other relevant information |
| --- |

1. **Details of information to be extracted from Clinical Guidelines**

| 1. **Identification features**  - Record number/code (to uniquely identify guideline) - Professional group or organization developing guideline (e.g. NICE, ADA, WHO, etc.) - Title - Citation - Source of Funding - Site (e.g. country) - Date produced - Date last updated      1. **General characteristics of clinical guideline**  - Aims/objectives - Type of clinical guideline (e.g. published, e-learning) - Clinical or health question(s) covered by the guideline (e.g. oral cancer, periodontal disease, cardiac conditions, etc.) - Target users of the guideline (general practitioners- medical or dental, specialists, etc.)  1. **Population/Participants**  - Patients, public, etc.- to whom the guideline is meant to apply - Age group - Gender - Socioeconomic status - Tobacco user, alcohol user, HPV/sexual behaviours, etc.   **Interventions**   - Preventive interventions (e.g. aim; behavioural/pharmacologic; length and content of intervention; delivered by whom; number of follow up sessions; referral pathways) - Risk factors assessed or focused on (e.g. tobacco, alcohol, HPV, etc.) - Method of risk factor assessment (e.g. how to ask about behaviour / assess risk) - Different options for assessment/prevention of the condition or health issue  1. **Outcomes/Recommendations**  - Key recommendations - Recommendations or best practice related to oral cancer risks, history taking, patient recall, delivery of preventive interventions, referral pathways, etc. - Role of combined interventions (e.g. multiple risks) - Role of communicating risks (e.g. identifying teachable moment) - Method and criteria for formulating the recommendations - Health benefits, side effects, and risks considered in formulating the recommendations - Link between the recommendations and the supporting evidence - **Settings/context** - Type of practice- Primary care setting- medical or dental  1. **Development/Presentation of clinical guideline**  - Method and criteria for selecting the evidence (e.g. sources consulted/searched- databases, organizations, health boards, etc.) - Guideline review procedure (e.g. externally reviewed by experts prior to its publication) - Procedure for updating the guideline - Strengths and limitations of the body of evidence - Advice and/or tools for dissemination/implementation of the guideline (e.g. a summary document, a quick reference guide, educational tools, results from a pilot test, patient leaflets, or computer support)   **Main results and authors conclusions**  **Summary points/Notes**  Any other relevant information |
| --- |
